# Stress hyperglycemia ratio and risk of incident myocardial infarction in the general population: a large-scale cohort study

**DOI:** 10.3389/fnut.2025.1601137

**Published:** 2025-07-03

**Authors:** Wenke Cheng, Xianlin Zhang, Jiqian Shi, Huaiyu Ruan, Pinfang Kang, Hongyan Sun, Meiyang Xu, Zhongyan Du, Bi Tang

**Affiliations:** ^1^Department of Cardiology, The First Affiliated Hospital of Bengbu Medical University, Bengbu, China; ^2^Department of Neurology, The First Affiliated Hospital of Bengbu Medical University, Bengbu, China; ^3^Zhejiang Key Laboratory of Blood-Stasis-Toxin Syndrome, Zhejiang Engineering Research Center for “Preventive Treatment” Smart Health of Traditional Chinese Medicine, School of Basic Medical Sciences, Zhejiang Chinese Medical University, Hangzhou, China

**Keywords:** stress hyperglycemia ratio, acute myocardial infarction, ST-segment elevation myocardial infarction, non-ST-segment elevation myocardial infarction, UK Biobank

## Abstract

**Background:**

Stress hyperglycemia ratio (SHR), which combines acute admission glucose with chronic glycemic indices, is a novel marker of stress hyperglycemia. Its association with acute myocardial infarction (AMI) risk in the general population remains unclear.

**Methods:**

This prospective cohort study used data from the UK Biobank and included 337,620 participants without known cardiovascular disease (CVD). SHR was calculated as admission glucose/[(28.7 × HbA1c%) – 46.7], with levels categorized into quintiles. The primary outcome was incident AMI, while ST-segment elevation myocardial infarction (STEMI) and non-ST-segment elevation myocardial infarction (NSTEMI) were evaluated as secondary outcomes. Cox proportional hazards models assessed the relationship between SHR and incident AMI risk. An accelerated failure time model was used to evaluate the effect of SHR on time to AMI onset, and dynamic changes in SHR were analyzed using a restricted cubic spline (RCS).

**Results:**

During a median follow-up of 164.8 months (IQR: 155.7–173.6), 10,598 AMI events, including 3,019 STEMI and 5,711 NSTEMI cases, were recorded. Compared with the fourth quintile, the first, second, and third quintiles had increased AMI risks by 19% (HR 1.19; 95% CI 1.12–1.27), 16% (HR 1.16; 95% CI 1.09–1.24), and 7% (HR 1.07; 95% CI 1.00–1.14), respectively, with no significant increase observed in the highest quintile. RCS analysis revealed a U-shaped relationship between SHR and incident AMI risk (P for non-linearity < 0.001), with the lowest risk at an SHR of 0.966.

**Conclusion:**

In the general population without known CVD, SHR exhibited a U-shaped association with incident AMI risk, with the lowest risk observed at an SHR of 0.966, particularly at levels below this threshold.

## Graphical Abstract

In this prospective UK Biobank cohort of 337,620 individuals without cardiovascular disease, SHR exhibited a U-shaped association with incident AMI risk, with the lowest risk at an SHR of 0.966 (*P* for nonlinearity < 0.001). Both low and high SHR levels were associated with elevated AMI risk. In addition, lower SHR levels were associated with earlier AMI onset, with the first quintile advancing the median AMI onset by up to 13.23 months compared to the fourth quintile. 
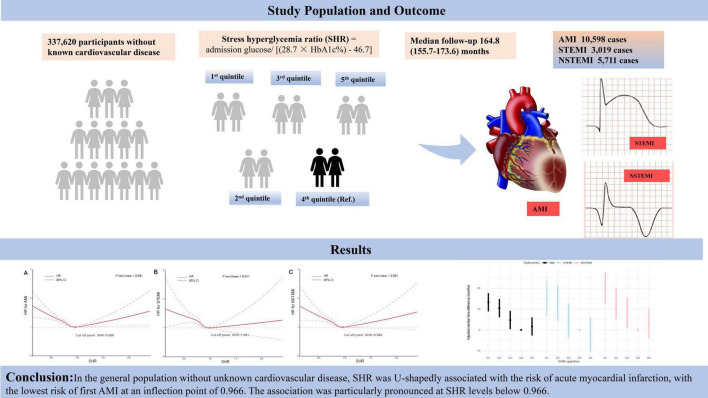

## Introduction

Acute myocardial infarction (AMI) is a major contributor to global mortality and morbidity, presenting substantial challenges to healthcare systems worldwide. With an annual incidence approaching 3 million cases globally, approximately 70% of AMI cases are attributed to atherosclerotic plaque rupture accompanied by thrombosis (1). AMI is categorized into non-ST-segment elevation myocardial infarction (NSTEMI) and ST-segment elevation myocardial infarction (STEMI) based on electrocardiographic characteristics. Despite advances in understanding AMI pathophysiology and management, the identification of effective biomarkers for risk stratification in the general population remains a central focus in cardiovascular research.

Stress hyperglycemia, characterized by transient elevations in blood glucose levels induced by acute illness or physiological stress, is a common adaptive response (2). It primarily results from increased secretion of counter-regulatory hormones such as catecholamines and cortisol, which promote gluconeogenesis and glycogenolysis, thereby raising blood glucose levels (3, 4). Moreover, stress hyperglycemia reflects the body’s response to severe illness through enhanced inflammatory and neurohormonal activation. It is often indicative of illness severity and serves as a critical marker for risk assessment in hospitalized patients (5–7). However, admission blood glucose levels alone may not fully capture the acute hyperglycemic state, as they are influenced by the patient’s underlying chronic glycaemic control (8). Furthermore, using a single glycemic threshold for risk assessment (e.g., 180 mg/dL) may misjudge individuals’ true risk, especially if chronic metabolic conditions are present (2, 9).

In this context, the stress hyperglycemia ratio (SHR)—which integrates acute admission blood glucose levels with chronic glycemic indices, such as glycated hemoglobin (HbA1c)—has emerged as a novel marker of stress-induced hyperglycemia (10). Although once considered a benign physiological adaptation, growing evidence highlights the detrimental effects of stress hyperglycemia, especially in patients with AMI (8, 11–13). Mechanistically, this may involve the promotion of oxidative stress and endothelial dysfunction, both of which can worsen myocardial injury and hinder recovery. Atherosclerosis, the pathological foundation of AMI, is driven by persistent inflammation, with oxidative stress playing a pivotal role in vascular dysregulation. Endothelial dysfunction, often preceding myocardial infarction and triggered by inflammation or infection, accelerates atherosclerotic progression, plaque instability, and thrombus formation—ultimately leading to AMI (14). Despite the biological plausibility, the relationship between SHR and AMI risk in the general population remains poorly characterized. We hypothesize that an elevated SHR, mediated by oxidative stress and endothelial dysfunction, may identify individuals at heightened risk for AMI. Therefore, this study aims to investigate the association between SHR and AMI risk in the general population.

## Materials and methods

### Study design and data source

This prospective cohort study utilized data from the UK Biobank, collected between 2006 and 2010 across various centers in the United Kingdom. The detailed methodology has been previously described (15). At baseline, comprehensive data were obtained on demographic and clinical characteristics, lifestyle factors, medical history, and biological samples through physical examinations, structured interviews, and laboratory assessments. Ethical approval for the study was granted by the North West Multi-Centre Research Ethics Committee (REC reference: 11/NW/0382), and written informed consent was obtained from all participants. Additional details are available on the UK Biobank website^1^.

A total of 384,159 participants with complete data on the SHR and no prior history of coronary artery disease were initially included. Participants who were pregnant (n = 111) or had a history of cancer (n = 40,850) were excluded. Furthermore, individuals with pre-existing othter cardiovascular conditions were excluded to minimize confounding effects, including those with heart failure (*n* = 542), valvular heart disease (*n* = 1,781), cardiomyopathy (*n* = 281), and arrhythmias (*n* = 754). After applying these exclusion criteria, the final analytic cohort comprised 337,620 participants. This study was conducted in accordance with the ethical standards of the Declaration of Helsinki.

### Assessment of SHR

Standard hematological tests were performed on fresh whole blood samples within 24 h of collection. Blood glucose levels were measured using Beckman Coulter AU5800 analyzers, while glycated hemoglobin A1c (HbA1c) was assessed using Bio-Rad Variant II Turbo analyzers. A quality control protocol was implemented to ensure the accuracy and reliability of HbA1c measurements. This involved bracketing participant samples with internal quality control materials at low, medium, and high concentrations. The precision of glucose measurements, as reflected by coefficients of variation (CV), ranged from 1.49 to 1.82%. For HbA1c, the CV ranged from 1.46 to 2.13%, indicating high measurement precision.

Estimated average glucose (eAG) levels were calculated from HbA1c values using the following equation: eAG (mg/dL) = 28.7 × HbA1c (%) − 46.7 (16). The SHR was then calculated by dividing the admission blood glucose level (mg/dL) by the eAG (mg/dL) (10), with the blood glucose value recorded at the initial assessment considered as the admission level.

### Assessment of other covariates

The baseline survey collected self-reported data on a range of variables, including age, sex, race, blood pressure, lipid profiles, physical activity levels, the Townsend Deprivation Index (TDI), chronic health conditions, medication use, fasting duration, and smoking and alcohol consumption patterns. The TDI, an established measure of socioeconomic status, incorporated indicators such as employment status, car and home ownership, and living space per person, with higher scores reflecting greater socioeconomic deprivation. To assess dietary risk factors, a methodology similar to that used in previous UK Biobank studies was employed to construct a composite dietary risk score (17). Briefly, nine dietary components—processed meats, red meats, fish, milk, butter/margarine, cereals, table salt, water, and fruits and vegetables—were selected for inclusion in the score. These components were categorized based on adherence to dietary guidelines recommended by UK and European health authorities. Points were assigned for consumption patterns deviating from these recommendations, yielding a total dietary score ranging from 0 (healthiest) to 9 (least healthy). Physical activity levels were quantified in total metabolic equivalent (MET) minutes per week, calculated using an adapted version of the International Physical Activity Questionnaire. Standardized protocols were followed to measure body mass index (BMI), blood pressure, and lipid profiles. Baseline comorbidities were ascertained through self-reported information obtained via questionnaires or interviews at enrolment, as well as through diagnostic codes from hospital records and surgical procedure data.

### Assessment of AMI and its subtypes

AMI and its subtypes were identified using algorithmically defined outcomes derived from the Health-Related Outcomes data within the UK Biobank. This algorithm-based classification system detects AMI events and subtypes with high accuracy by integrating coded health information from multiple sources, including baseline assessments, hospital admission records, and death registries. This automated approach is particularly beneficial in large-scale epidemiological research, as it streamlines outcome ascertainment and reduces reliance on manual diagnostic code integration. AMI diagnoses were coded according to the International Classification of Diseases, Ninth and Tenth Revisions (ICD-9 and ICD-10) (Supplementary Table S1).

The algorithms, developed by the UK Biobank Outcome Adjudication Group, are designed to maximize positive predictive value (PPV) for health event identification. A systematic review by the UK Biobank Cardiac Outcomes Group reported PPVs ranging from 75 to 100% for algorithmically defined AMI based on linked hospital admission records, with PPVs exceeding 90% for NSTEMI and ranging from 71 to 100% for STEMI (18). The PPV for AMI events identified from death registry data was approximately 70–75%.

The primary outcome of this study was incident AMI, with secondary outcomes including incident STEMI and NSTEMI. For each participant, the observation period extended from the date of enrollment to the earliest occurrence of incident AMI, death, or the censoring date of 29 November 2022.

### Statistical analysis

Missing categorical variables were handled using missing indicator methods, while continuous variables were imputed using the mean. The distribution of continuous variables was assessed using the Kolmogorov–Smirnov test, which indicated non-normality. Consequently, categorical variables were summarized as frequencies and percentages, and continuous variables were reported as medians with interquartile ranges (IQRs). SHR levels were stratified into quintiles. Group comparisons were performed using the Chi-squared test for categorical variables and the Kruskal–Wallis test for continuous variables. AMI incidence within SHR quintiles was expressed as events per 1,000 person-years, while cumulative incidence was calculated as the number of events divided by the total population at risk.

Kaplan–Meier survival curves were generated to estimate AMI incidence across SHR quintile groups, and differences between groups were assessed using the log-rank test. Based on the observed exposure–response pattern, the fourth SHR quintile was selected as the reference category. Hazard ratios (HRs) and 95% confidence intervals (CIs) for the association between SHR and incident AMI were estimated using Cox proportional hazards models. The proportional hazards assumption was verified using Schoenfeld residuals, with no significant violations observed.

Potential confounders were identified based on established *a priori* knowledge relevant to causal inference. A directed acyclic graph (DAG) was constructed using the DAGitty online tool^2^ to determine the minimally sufficient adjustment set. This set included sex, age, race, BMI,TDI, fasting duration, physical activity, dietary score, diabetes mellitus, insulin use, and smoking and alcohol consumption status (Supplementary Figure S1). Hypertension, antihypertensive medication, blood pressure and lipid parameters, which are potential mediating variables in the exposure-outcome association, were not adjusted for in the main analysis. Three Cox regression models were fitted: Model 1 was unadjusted (crude); Model 2 adjusted for age, sex, and race; and Model 3 additionally adjusted for the full set of confounding variables identified through the DAG.

To explore potential non-linear associations between SHR and incident AMI risk, restricted cubic spline (RCS) functions were fitted with four knots placed at the 5th, 35th, 65th, and 95th percentiles of the SHR distribution. The selection and placement of knots followed Harrell’s recommended strategy (19), balancing model flexibility and avoidance of overfitting, a method widely adopted in large-scale epidemiological studies (20–23). Non-linearity was tested using a log-likelihood ratio test. Where a significant non-linear association was observed, a two-piecewise linear regression model was applied to estimate the inflection point. Further analyses are detailed in Supplementary Appendix 1. Additionally, SHR values were standardized using Z-score normalization (mean = 0, SD = 1) to quantify the change in AMI risk per one standard deviation increase in SHR before and after the inflection point. Subgroup analyses were conducted across predefined strata, including age (< 55 vs. ≥ 55 years), sex (men vs. women), race (Caucasian vs. non-Caucasian), BMI (< 30 vs. ≥ 30 kg/m^2^), and diabetes status (yes vs. no). Between-group interaction *P* values were obtained using likelihood ratio tests.

To assess the temporal influence of SHR on AMI onset, an accelerated failure time (AFT) model was fitted, under the assumption that covariates accelerate or delay event timing independently of the proportional hazards assumption. A multivariate AFT model evaluated the time to AMI onset across SHR quintiles, using the fourth quintile (Q4) as the reference group. Differences in median AMI onset time were computed in months by subtracting the reference value from each comparison group. Negative values indicated delayed AMI onset, while positive values indicated earlier onset relative to Q4. A flexible Weibull distribution was used to accommodate the right-skewed distribution of time-to-event data (Supplementary Figures S2,S3).

Sensitivity analyses were conducted to assess the robustness of the findings using two approaches. First, multiple imputation was applied to address missing data, using predictive mean matching across five replicates and Markov Chain Monte Carlo methods. Imputed datasets were analyzed using Cox models, and results were pooled accordingly. Second, participants who experienced AMI within 2 years of enrolment were excluded to minimize potential reverse causality. Third, the sample was restricted to participants with SHR values between the 2.5th and 97.5th percentiles to minimize the influence of extreme values and assess whether the association between SHR and AMI remained robust. Fourth, the Cox models were further adjusted for hypertension, antihypertensive medication use, and lipid-lowering therapy to assess whether the association remained after accounting for these potential mediators. All statistical analyses were performed using R software (version 4.2.0), and a two-sided *P* < 0.05 was considered statistically significant.

## Results

**FIGURE 1:**
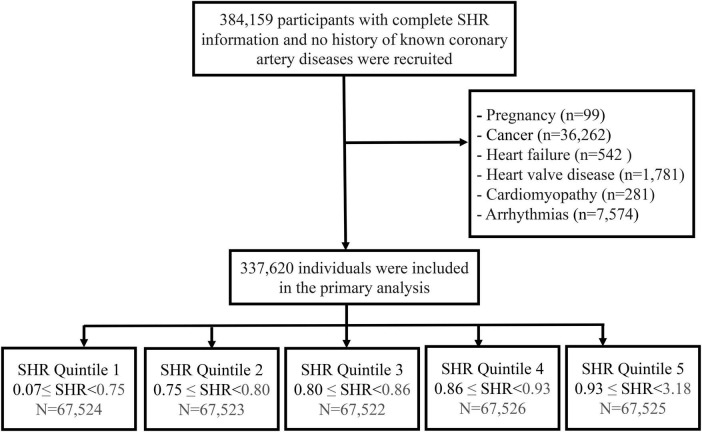
Flow chart of the study.

Figure 1 presents the participant selection flowchart. Table 1 summarizes the baseline characteristics of the 337,620 participants without a history of CVD. The median age of the cohort was 57 years (interquartile range [IQR]: 49–62 years), with 45.72% being male and 94.26% identified as Caucasian. Participants were stratified into quintiles according to their SHR levels, with the following ranges: 0.07– < 0.75, 0.75– < 0.80, 0.80– < 0.86, 0.86– < 0.93, and 0.93–3.18. Significant differences across SHR quintiles were observed for multiple baseline variables, including age, sex, race, TDI, BMI, blood pressure, lipid profiles, physical activity levels, chronic health conditions, medication use, fasting duration, and smoking and alcohol consumption behaviors.

**TABLE 1:** Baseline characteristics of 337,620 participants stratified by quintiles of stress hyperglycemia ratio.

Variables	Total	SHR quintiles					
		Q1 (0.07– < 0.75)	Q2 (0.75–<0.80)	Q3 (0.80–< 0.86)	Q4 (0.86– < 0.93)	Q5 (0.93–3.18)	*P*-value
Number	337,620	67,524	67,523	67,522	67,526	67,525	
Age (years)	57 (49–62)	57 (50–63)	57 (50–63)	57 (49–62)	56 (48–62)	56 (48–62)	< 0.001
Men (%)	154,347 (45.72%)	31,459 (46.59%)	29,221 (43.28%)	29,644 (43.90%)	30,949 (45.83%)	33,074 (48.98%)	< 0.001
Caucasian (%)	318235 (94.26%)	61138 (90.54%)	63376 (93.86%)	64301 (95.23%)	64788 (95.95%)	64632 (95.72%)	< 0.001
TDI	−2.17 (−3.66–0.45)	−2 (−3.59–0.86)	−2.17 (−3.67–0.42)	−2.26 (−3.69–0.28)	−2.21 (−3.68–0.30)	−2.17 (−3.65–0.40)	< 0.001
BMI (Kg/m^2^)	26.66 (24.08–29.73)	26.97 (24.19–30.34)	26.66 (24.06–29.72)	26.51 (24.01–29.48)	26.46 (24–29.37)	26.74 (24.16–29.78)	< 0.001
DBP (mmHg)	82 (75.50–89)	81.50 (75–88.50)	82 (75.50–89)	82.50 (75.50–89)	82.50 (76–89.50)	82.50 (76–89.50)	< 0.001
SBP (mmHg)	136 (124.50–149)	134.50 (123.5–147.5)	135.50 (124–148.5)	136 (124.50–149)	136.50 (125–149.5)	138 (126.50–151)	< 0.001
Physical activity (MET-min/week)	2626.50 (1070–2906)	2661 (1034–2825.25)	2661 (1074–2910)	2661 (1097–2977.5)	2593 (1095–2968.5)	2535 (1053–2868)	< 0.001
Diet score	5 (4–6)	5 (4–6)	5 (4–6)	5 (4–6)	5 (4–6)	5 (4–6)	< 0.001
HbA1c (%)	5.36 (5.13–5.59)	5.60 (5.39–5.84)	5.45 (5.28–5.64)	5.34 (5.18–5.52)	5.22 (5.06–5.42)	5.11 (4.89–5.39)	< 0.001
TC (mmol/L)	5.71 (5–6.46)	5.68 (4.93–6.46)	5.78 (5.07–6.53)	5.77 (5.08–6.51)	5.72 (5.02–6.46)	5.59 (4.87–6.34)	< 0.001
HDL-C (mmol/L)	1.40 (1.20–1.70)	1.40 (1.10–1.60)	1.40 (1.20–1.70)	1.40 (1.20–1.70)	1.40 (1.20–1.70)	1.40 (1.20–1.70)	< 0.001
LDL-C (mmol/L)	3.57 (3.01–4.15)	3.56 (2.98–4.16)	3.62 (3.07–4.21)	3.61 (3.07–4.19)	3.57 (3.03–4.14)	3.47 (2.92–4.04)	< 0.001
TG (mmol/L)	1.47 (1.04–2.13)	1.57 (1.09–2.28)	1.48 (1.05–2.13)	1.43 (1.03–2.06)	1.41 (1–2.03)	1.47 (1.02–2.17)	< 0.001
Glucose (mg/dl)	88.49 (82.64–95.24)	79.36 (74.05–84.35)	85.30 (81.41–89.41)	88.52 (84.60–92.81)	91.84 (87.46–96.66)	99.79 (92.75–110.92)	< 0.001
Fasting time (hours)	3 (2–4)	3 (2–4)	3 (3–5)	3 (3–5)	3 (3–4)	3 (2–4)	< 0.001
Diabetes mellitus (%)	14199 (4.21%)	4951 (7.33%)	1703 (2.52%)	1393 (2.06%)	1461 (2.16%)	4691 (6.95%)	< 0.001
Hypertension (%)	83465 (24.72%)	18034 (26.71%)	15949 (23.62%)	15512 (22.97%)	15685 (23.23%)	18285 (27.08%)	< 0.001
Current smoker (%)	35381 (10.49%)	10351 (15.35%)	7700 (11.42%)	6456 (9.57%)	5811 (8.61%)	5063 (7.51%)	< 0.001
Current drinker (%)	311232 (92.28%)	60411 (89.57%)	61936 (91.82%)	62682 (92.92%)	63268 (93.78%)	62935 (93.33%)	< 0.001
Antihypertensives use (%)	58110 (17.21%)	13037 (19.31%)	11012 (16.31%)	10482 (15.52%)	10601 (15.70%)	12978 (19.22%)	< 0.001
Lipid-lowering drugs use (%)	44360 (13.14%)	11840 (17.53%)	8730 (12.93%)	7513 (11.13%)	7226 (10.70%)	9051 (13.40%)	< 0.001
Insulin use (%)	2796 (0.83%)	1071 (1.59%)	187 (0.28%)	182 (0.27%)	188 (0.28%)	1168 (1.73%)	< 0.001

TDI, Townsend deprivation index; BMI, body mass index; TC, total cholesterol; LDL-C, low-density lipoprotein cholesterol; HDL-C, high-density lipoprotein; MET, metabolic equivalent task; SBP, systolic blood pressure; DBP, diastolic blood pressure.

### SHR and incident AMI

**FIGURE 2:**
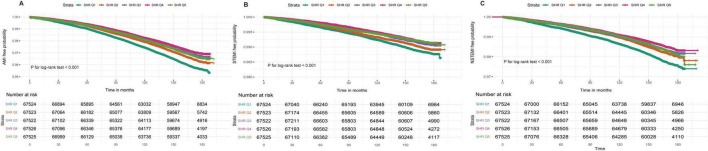
**(A)** Kaplan-Meier survival curves for AMI incidence in the SHR quintiles groups. **(B)** Kaplan-Meier survival curves for STEMI incidence in the SHR quintile groups. **(C)** Kaplan-Meier survival curves for NSTEMI incidence in the SHR quintile groups SHR, stress hyperglycemia ratio. AMI, acute myocardial infarction; STEMI, ST-segment elevation myocardial infarction; NSTEMI, non-ST-segment elevation myocardial infarction.

During a median follow-up of 164.8 months (IQR: 155.7–173.6), 10,598 cases of AMI (3.14% of the cohort), 3,019 cases of STEMI (0.89%), and 5,711 cases of NSTEMI (1.69%) were documented. Kaplan–Meier survival analysis revealed significant differences in the incidence of AMI, STEMI, and NSTEMI across SHR quintiles (log-rank test, *P* < 0.001), with the lowest incidence observed in the fourth quintile and the highest in the first. A similar trend was noted for cumulative incidence (Figure 2 and Table 2).

**TABLE 2:** Cox proportional hazards analysis to assess SHR quintile levels and risk of AMI.

	Event/total	Person-years	Incidence rate (Per 1,000 perso*n*-years)	Unadjusted HR (95%CI)	*P*-value	Adjusted HR^†^ (95%CI)	*P*-value	Multivariate adjusted HR^‡^ (95%CI)	*P*-value
AMI	10,598/337,620								
SHR Q1	2,693/67,524	902,129.04	2.98	1.46 (1.37–1.55)	< 0.001	1.38 (1.30–1.47)	< 0.001	1.19 (1.12–1.27)	< 0.001
SHR Q2	2,214/67,523	903,592.23	2.45	1.22 (1.15–1.30)	< 0.001	1.21 (1.14–1.29)	< 0.001	1.16 (1.09–1.24)	< 0.001
SHR Q3	1,961/67,522	902,435.54	2.17	1.08 (1.02–1.16)	0.013	1.08 (1.02–1.16)	0.014	1.07 (1.0–1.14)	0.037
SHR Q4	1,800/67,526	899,636.56	2	Reference		Reference		Reference	
SHR Q5	1,984/67,525	895,087.93	2.22	1.11 (1.04–1.18)	0.002	1.09 (1.02–1.16)	0.012	1.02 (0.96–1.09)	0.453
STEMI	3,019/337,620								
SHR Q1	750/67,524	910,709.49	0.82	1.42 (1.27–1.59)	< 0.001	1.36 (1.22–1.52)	< 0.001	1.21 (1.08–1.36)	< 0.001
SHR Q2	654/67,523	910,754.18	0.72	1.24 (1.10–1.39)	< 0.001	1.25 (1.11–1.40)	< 0.001	1.19 (1.06–1.34)	0.003
SHR Q3	564/67,522	908,890.51	0.62	1.07 (0.95–1.20)	0.266	1.08 (0.96–1.21)	0.215	1.06 (0.94–1.20)	0.31
SHR Q4	525/67,526	905,460.19	0.58	Reference		Reference		Reference	
SHR Q5	526/67,525	901,436.54	0.58	1.01 (0.89–1.14)	0.912	0.98 (0.87–1.10)	0.721	0.97 (0.86–1.09)	0.61
NSTEMI	5,711/337,620								
SHR Q1	1,463/67,524	908,619.47	1.61	1.52 (1.40–1.65)	< 0.001	1.44 (1.33–1.56)	< 0.001	1.24 (1.14–1.34)	< 0.001
SHR Q2	1,167/67,523	909,159.64	1.28	1.22 (1.12–1.33)	< 0.001	1.20 (1.10–1.31)	< 0.001	1.15 (1.06–1.26)	0.001
SHR Q3	1,065/67,522	906,998.69	1.17	1.12 (1.02–1.22)	0.014	1.11 (1.02–1.21)	0.017	1.10 (1.01–1.20)	0.036
SHR Q4	950/67,526	903,990.77	1.05	Reference		Reference		Reference	
SHR Q5	1,066/67,525	899,995.58	1.18	1.13 (1.03–1.23)	0.007	1.11 (1.01–1.21)	0.024	1.04 (0.95–1.13)	0.442

SHR, stress hyperglycemia ratio; AMI, acute myocardial infarction; STEMI, ST-segment elevation myocardial infarction; NSTEMI, non-ST-segment elevation myocardial infarction.

^†^Indicates model adjusted for age, sex, and race. ^‡^Indicates model adjusted for age, sex, race, body mass index, Townsend Deprivation Index, physical activity, diet score, insulin use, fasting time, diabetes mellitus, smoking and drinking status.

Cox regression analyses were performed using the fourth SHR quintile as the reference group, with results presented in Table 2. In unadjusted models, both higher and lower SHR quintiles were associated with increased risks of AMI and NSTEMI (*P* < 0.05), while lower SHR levels were associated with higher STEMI risk (*P* < 0.05). These associations persisted after adjusting for age, sex, and race. In the fully adjusted multivariable model, the first, second, and third SHR quintiles were associated with a 19% (HR 1.19, 95% CI: 1.12–1.27), 16% (HR 1.16, 95% CI: 1.09–1.24), and 7% (HR 1.07, 95% CI: 1.00–1.14) increased risk of AMI, respectively. For STEMI, the corresponding risk increases were 21% (HR 1.21, 95% CI: 1.08–1.36), 19% (HR 1.19, 95% CI: 1.06–1.34), and 6% (HR 1.06, 95% CI: 0.94–1.20). For NSTEMI, the increases were 24% (HR 1.24, 95% CI: 1.14–1.34), 15% (HR 1.15, 95% CI: 1.06–1.26), and 10% (HR 1.10, 95% CI: 1.01–1.20), respectively. Notably, the highest SHR quintile was not associated with a significantly increased risk of AMI compared with the fourth quintile.

### SHR and time to AMI onset

**FIGURE 3:**
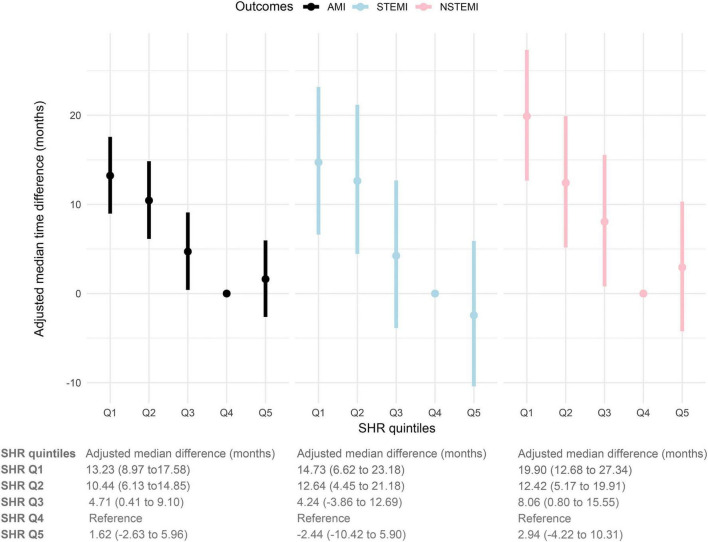
Multivariate accelerated failure time models assessed the time-adjusted median difference in the incidence of AMI, STEMI, and NSTEMI in the first, second, third, and highest quintiles compared to the fourth quintile of the SHR. Adjusted median difference = median occurrence time in reference group (Q1)—median occurrence time in comparison group. Negative values indicate a delay in the onset of events, while positive values indicate an earlier onset.

In the multivariate AFT model, compared to the fourth SHR quintile, AMI, STEMI and NSTEMI onset was advanced in the first, second and third SHR quintiles (Figure 3). Specifically, the adjusted median time to incident AMI was shortened by 13.23, 10.44, and 4.71 months in the first, second, and third SHR quintile groups, respectively, compared to the fourth quintile. Moreover, the adjusted median time of onset of STEMI was advanced by 14.73, 12.64, and 4.24 months, respectively, while that of NSTEMI was advanced by 19.90, 12.42 and 8.06 months, respectively. However, the time to AMI, STEMI and NSTEMI was not significantly advanced in the highest SHR quintile compared with the fourth quintile.

### Exposure-effect relationship between SHR and incident AMI risk

**FIGURE 4:**
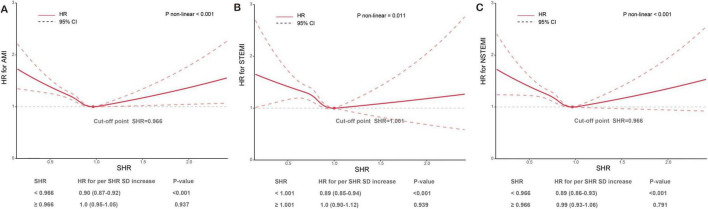
Restricted cubic splines assessed SHR levels with the risk of incident AMI **(A)**, STEMI **(B)**, and NSTEMI **(C)**. SHR, stress hyperglycemia ratio; AMI, acute myocardial infarction; STEMI, ST-segment elevation myocardial infarction; NSTEMI, non-ST-segment elevation myocardial infarction. The figure demonstrates the U-shaped relationship between SHR levels and the risk of incident AMI **(A)**, STEMI **(B)**, and NSTEMI **(C)**. The cut-off point represents the lowest risk level, where the SHR value results in the minimum hazard. The analysis explores the hazard within two SHR intervals—below and above the cut-off point—by examining the hazard ratio (HR) for each standard deviation (SD) increase in SHR levels.

The RCS analysis revealed a non-linear relationship between SHR levels and incident AMI risk, which showed a U-shaped curve (P for non-linear < 0.001) (Figure 4). When analyzing SHR with the risk of AMI, STEMI and NSTEMI, their inflection points were found at 0.966, 1.001, and 0.966, respectively. As the SHR level increased, the risk of AMI initially decreased significantly but then gradually increased. Specifically, when SHR was < 0.966, each SD increase in SHR was associated with a 10 and 11% reduction in the risk of AMI and NSTEMI, respectively. However, when SHR was ≥ 0.966, no further increase in AMI or NSTEMI risk was observed per SD increase in SHR. A similar pattern was seen for STEMI: when SHR was < 1.001, the risk decreased by 11% per SD increase, while no additional risk change was observed beyond this threshold.

**FIGURE 5:**
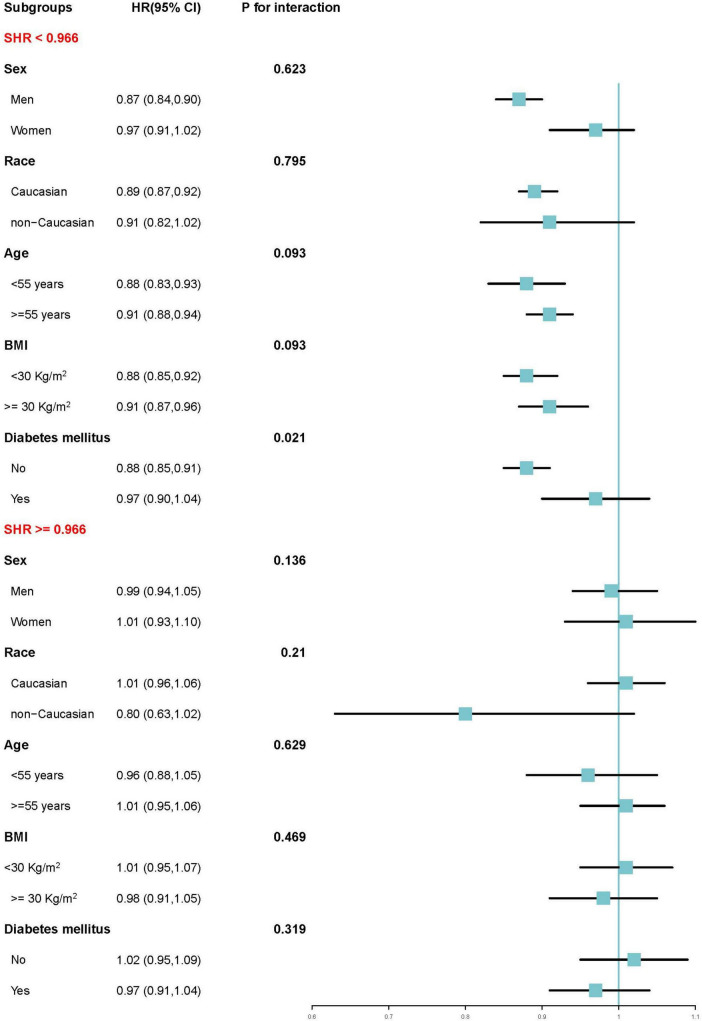
Subgroup analyses assessed changes in increased AMI risk per one standard deviation before and after the inflection point (SHR = 0.966) according to different clinical characteristics. SHR, stress hyperglycemia ratio; AMI, acute myocardial infarction.

Given this non-linear association, subgroup analyses were conducted to assess the effect of each SD increase in SHR on AMI risk before and after the inflection point of 0.966 across various strata. As shown in Figure 5, in the SHR range < 0.966, AMI risk consistently declined across subgroups of sex, race, age, and BMI, with no significant interactions. However, a modest interaction was observed in the diabetes mellitus subgroup, where the risk reduction was more pronounced among individuals without diabetes. In the SHR range ≥ 0.966, no significant increase in AMI risk was found in any subgroup, and no interactions were detected.

### Sensitivity analyses

In the sensitivity analyses, results obtained through multiple imputation were consistent with those from the primary analyses (Supplementary Table S2). Similarly, excluding participants who experienced AMI within the first 2 years of follow-up did not materially alter the findings, further confirming the robustness of our results (Supplementary Table S3). Consistent results were also observed when restricting the analysis to participants with SHR values between the 2.5th and 97.5th percentiles, minimizing the influence of extreme values (Supplementary Table S4). In addition, further adjustment for hypertension status, antihypertensive medication use, and lipid-lowering therapy did not materially change the association between SHR and AMI risk (Supplementary Table S5).

## Discussion

In this large prospective cohort study, we identified a U-shaped association between the SHR and the incidence of AMI in individuals without pre-existing CVD, with a threshold inflection point at 0.966. SHR levels both below and above this threshold were associated with an increased risk of AMI and earlier onset of the disease, with particularly pronounced effects observed in the lower SHR range.

Previous clinical epidemiological studies have extensively explored the role of stress hyperglycemia in predicting the prognosis of patients with AMI, consistently finding that elevated stress hyperglycemia levels correlate with higher risks of adverse cardiovascular events and mortality (24–27). Although the precise mechanisms linking stress hyperglycemia to adverse outcomes remain incompletely understood, it likely reflects the severity of acute events and suboptimal glycemic control (8). Stress hyperglycemia may exacerbate acute cardiac events through various pathways, including microvascular obstruction, reduced endothelium-dependent vasodilatory function, impaired platelet nitric oxide reactivity and exacerbation of vascular injury induced by hyperglycemia. Additionally, insulin resistance has been implicated as a contributing factor in stress hyperglycemia (28). Recently, Whitlock et al. demonstrated that the regulation of hepatic gluconeogenesis involving the forkhead box protein O transcription factor may play a crucial role in stress hyperglycemia (29).However, admission blood glucose levels alone may not accurately reflect true stress-induced hyperglycemia, necessitating consideration of long-term chronic blood glucose levels, which can be determined by the following formula ([(28.7 × HbA1c [%]) − 46.7]) (16). Therefore, SHR has emerged as a more comprehensive biomarker than admission hyperglycemia alone (10), supported by several recent observational studies (2, 7, 8, 30). Nevertheless, the association between SHR and AMI risk in the general population remains unclear. The pathophysiological link between the brain and heart forms the basis of CVD, particularly AMI. Activation of the hypothalamic-pituitary axis and the sympathetic-adrenal system in response to stress leads to increased release of epinephrine, norepinephrine and pro-inflammatory cytokines (e.g., TNF-α, IL-1 and IL-6) (31). This hyperactivity of the sympathetic nervous system has been associated with the development of various CVD, including hypertension, arrhythmias, atherosclerosis, heart failure and AMI (32).

Preclinical studies indicate that chronic stress-induced release of norepinephrine is associated with endothelial dysfunction and atherosclerosis development, contributing to oxidative stress and inflammation. Normally, alpha-adrenergic-mediated vasoconstriction does not significantly affect coronary blood flow, but in certain pathological states, such as endothelial dysfunction and atherosclerosis, the blood flow is affected and results in myocardial ischemia (33). Notably, the excitation of sympathetic afferent nerves in the left ventricle is speculated to be an important mechanism of sympathetic activation after AMI. Furthermore, inflammation and activation of the immune system play a key role in the development and progression of coronary artery disease. Elevated pro-inflammatory cytokine levels are implicated in AMI risk, with certain pro-inflammatory cytokines such as IL-8 being associated with a high risk of coronary artery disease (34, 35).

The dose-dependent analyses elucidated a U-shaped relationship between SHR levels and AMI risk, with both low and high SHR associated with a higher risk of AMI. For the secondary outcomes, we explored two subtypes of AMI, namely STEMI and NSTEMI. STEMI is typically induced by complete blockage due to coronary thrombosis, whereas NSTEMI is induced by partial blockage or intermittent blockage due to flow instability. Consistent with the association between SHR and AMI risk, this U-shaped relationship persisted in the secondary outcome analyses of STEMI and NSTEMI, further confirming the stability and prevalence of this relationship between SHR and AMI. Unexpectedly, although a U-shaped association between the SHR and the risk of incident AMI was observed, the inverse relationship was more pronounced at lower SHR levels (< 0.966). This finding appears to contrast with most existing literature and prevailing clinical expectations (36–39). SHR provides a measure of stress-induced hyperglycemia, comparing current glucose levels with long-term glycemic control estimated from HbA1c. Stress is not inherently harmful but rather functions as a double-edged sword. Moderate stress responses mobilize metabolic reserves, maintain glucose supply, and stabilize hemodynamics, thereby offering short-term protective effects (40). Conversely, both insufficient and excessive stress responses can disrupt metabolic homeostasis and elevate cardiovascular risk. As a marker of the deviation between acute random glucose and chronic HbA1c, SHR serves as a proxy for the body’s glycemic adaptability under stress (40). Under stress, counterregulatory hormones such as adrenaline, cortisol, and glucagon are typically elevated to increase blood glucose, facilitating the body’s physiological response (41). Most previous studies linking elevated SHR to adverse outcomes have focused on populations with acute cardiovascular events or critical illness, where a high SHR is often interpreted as a marker of disease severity and poor prognosis. In contrast, evidence from community-based populations without baseline CVD is scarce. These discrepancies may arise from the fundamentally different metabolic implications of SHR across populations.

Consistent with our findings, Zhang et al. recently reported an inverse association between low SHR and CVD risk in older adults without baseline CVD (42). They identified a non-linear relationship with an inflection point at 0.985, closely aligning with our identified threshold of 0.966. The slightly higher cutoff in their study may be attributed to a broader CVD definition, which encompassed both heart disease and stroke. Similarly, Tan et al. reported a U-shaped association between SHR and cardiovascular mortality in individuals at CKM stages 0–3, a population without diagnosed CVD. Interestingly, no significant association was observed between higher SHR and cardiovascular mortality. They speculated that individuals with elevated SHR may possess greater metabolic reserves and stress-regulation capacity, thereby mitigating its harmful effects (43). These observations suggest that low SHR may reflect a high-risk phenotype characterized by impaired stress responsiveness or diminished metabolic reserve. Notably, individuals with low SHR often exhibit elevated HbA1c alongside relatively low random glucose levels, indicating chronic hyperglycemia without an adequate glycemic surge during stress (42). This “blunted stress response” may signal sympathetic nervous system dysfunction or impaired pancreatic reserve—features of reduced metabolic adaptability linked to heightened cardiovascular vulnerability (40). In contrast, among individuals without CVD, a moderately elevated SHR may reflect a transient and adaptive hormonal response, unlikely to result in long-term harm. In patients with established CVD or critical illness, however, a high SHR is more likely to represent maladaptive physiological stress, systemic inflammation, and metabolic dysregulation (40, 44). These individuals commonly exhibit chronic inflammation, β-cell dysfunction, and overactivation of the sympathoadrenal axis. In such contexts, a markedly elevated SHR may signal greater disease severity, enhanced inflammatory burden, and impaired glucose regulation—contributing to cardiovascular events via mechanisms including increased myocardial oxygen demand, platelet activation, and endothelial dysfunction. Taken together, these findings suggest that the prognostic significance of SHR may vary across populations depending on whether it reflects an adaptive or maladaptive stress response. This conceptual distinction may help reconcile inconsistencies in the literature and underscores the importance of context when interpreting the clinical relevance of SHR. Our results also reinforce the established role of glucose management in mitigating AMI risk. Subgroup analyses demonstrated consistent associations across most strata, with stronger associations observed among individuals without diabetes. This finding aligns with previous studies suggesting that SHR may serve as a more sensitive marker of adverse outcomes in non-diabetic individuals (26). The direct effect of diabetes on AMI risk may attenuate the relative contribution of SHR in diabetic populations.

The observed U-shaped association is unlikely to be explained by residual confounding, reverse causality, or measurement bias. Similar non-linear associations between SHR and adverse outcomes have been reported in multiple prior studies (8, 45–48), supporting the robustness of this pattern. While residual confounding cannot be entirely excluded, our covariate selection was guided by DAGs and prior evidence, reducing the risk of over adjustment and enhancing model stability. Sensitivity analyses further supported the robustness of the findings. Additional adjustment for hypertension, antihypertensive medication use, and lipid-lowering therapy—known risk factors for AMI—did not materially alter the effect estimates. To reduce the potential for reverse causation, individuals who experienced AMI events within the first 2 years of follow-up were excluded, thereby strengthening the temporal validity of our findings. Furthermore, fasting duration was adjusted for in all multivariable models to address potential bias from non-fasting glucose measurements.

Although the phenotypic trend between SHR and adverse outcomes appears consistent across studies, the identified cutoff values vary to some extent. Yang et al. also reported a U-shaped relationship between SHR and adverse cardiovascular events in patients with AMI, with an inflection point at 0.78 (8). In our study, the lowest risk of incident AMI was observed at an SHR of 0.966, suggesting a higher threshold in the general population compared to individuals with established AMI. Several factors may account for this discrepancy. First, population-related differences likely contribute to the variation. Yang et al. examined hospitalized patients with acute AMI, representing a secondary prevention setting. These individuals were under peak physiological stress, and the lower threshold may reflect heightened vulnerability to stress hyperglycemia during the acute phase. In contrast, our study was based on the UK Biobank cohort, consisting of generally healthy, middle-aged and older adults without known CVD. The objective was to evaluate SHR as a predictive marker for AMI risk in a primary prevention context. The differences in population characteristics and clinical setting likely explain the higher threshold observed in our analysis. Second, variations in glucose measurement conditions may also contribute. In Yang et al.’s study, SHR was derived from glucose levels obtained at hospital admission, reflecting acute illness and intensified stress responses. In contrast, glucose in the UK Biobank was primarily measured in non-fasting, outpatient settings, capturing more moderate physiological states. These contextual differences may shift the overall SHR distribution. Third, differences in statistical methodology should be considered. Yang et al. determined the optimal cutoff using the Youden Index from receiver operating characteristic curve analysis. In contrast, our study used restricted cubic spline modeling to examine non-linear associations, with the threshold defined at the nadir of AMI risk. These differing analytical approaches may have also contributed to the observed differences in cutoff values.

### Strength and limitations

To the best of our knowledge, this study is the first to investigate the association between SHR levels and AMI risk in the general population. The robust sample size of over 300,000 participants and the prospective cohort design enhances the validity and generalizability of the findings, aligning them more closely with real-world scenarios. Additionally, the incorporation of the AFT model, which analyses both the risk and timing of AMI onset, reinforces the stability of the results.

Nonetheless, there remain certain limitations. Firstly, the observational design of the study precludes the establishment of causal relationships between SHR and AMI risk. Secondly, despite adjusting for potential confounding and mediating variables identified through DAGs, residual confounding may persist, impacting result accuracy. Third, the use of non-fasting blood glucose levels for SHR calculation—due to logistical constraints in obtaining fasting samples in large cohorts—may introduce variability and affect the accuracy of SHR measurements. Although fasting time was adjusted for in the analyses, residual impact on the results cannot be entirely excluded. Finally, the study population was predominantly Caucasian individuals from the UK Biobank, limiting the generalizability of the findings to other ethnicities.

## Conclusion

In the general population without known CVD, SHR showed a U-shaped association with the risk of incident AMI, with the lowest risk at an inflection point of 0.966. The association was particularly pronounced at SHR levels below 0.966.

## Data Availability

The datasets presented in this study can be found in online repositories. The names of the repository/repositories and accession number(s) can be found in the article/Supplementary material.

## Footnotes

1
www.ukbiobank.ac.uk

2
www.dagitty.net
