# Supplementary material for: Stress hyperglycemia ratio and risk of incident myocardial infarction in the general population: a large-scale cohort study
Source: Front Nutr. 2025 Jul 3;12:1601137. doi: 10.3389/fnut.2025.1601137 (PMC12269421; doi:10.3389/fnut.2025.1601137)
Supplement: Supplementary file 1 [file Data_Sheet_1.pdf]

Supplement

Table S1. AMI Codes for UK Biobank Diagnostics.

Table S2. Multivariate COX regression analyses of SHR quintiles and the risk of AMI with multiple imputation of five datasets and then pooled.

Table S3. Multivariate COX regression analyses of SHR quintiles and the risk of incident AMI after excluding the participants who experienced AMI within two years of enrolment.

Table S4. Multivariate Cox regression analysis of SHR quintiles and incident AMI risk within the 2.5^{th}–97.5^{th} percentile range of SHR

Table S5. Multivariable Cox regression of SHR quintiles and incident AMI risk with additional adjustment for hypertension, antihypertensive use, and lipid-lowering therapy.

Figure S1. Directed acyclic graph of the link between SHR and the incidence of AMI.

Figure S2. Histogram of the distribution of Time to AMI.

Figure S3. Normal Q-Q Plot of time to AMI.

Table S1. AMI Codes for UK Biobank Diagnostics.

Code Type	Field Code 20002	Biobank Code Text	STEMI	NSTEMI	MI
UK Biobank Self Report	1075	Heart attack/myocardial infarction			✓
		**ICD 9 Codes**			
**Code Type**	**ICD 9 Code**	**ICD 9 Text**	**STEMI**	**NSTEMI**	**MI**
ICD 9 Code	410	Acute myocardial infarction	✓		✓
ICD 9 Code	410.0	Acute myocardial infarction of anterolateral wall	✓		✓
ICD 9 Code	410.1	Acute myocardial infarction of other anterior wall	✓		✓
ICD 9 Code	410.2	Acute myocardial infarction of inferolateral wall	✓		✓
ICD 9 Code	410.3	Acute myocardial infarction of infer posterior wall	✓		✓
ICD 9 Code	410.4	Acute myocardial infarction of other inferior wall	✓		✓
ICD 9 Code	410.5	Acute myocardial infarction of other lateral wall	✓		✓
ICD 9 Code	410.6	True posterior wall infarction	✓		✓
ICD 9 Code	410.7	Subendocardial infarction		✓	✓
ICD 9 Code	410.8	Acute myocardial infarction of other specified sites	✓		✓
ICD 9 Code	410.9	Acute myocardial infarction of unspecified site	✓		✓
ICD 9 Code	411.0	Post myocardial infarction syndrome			✓
ICD 9 Code	412.X[4]	Old myocardial infarction			✓
ICD 9 Code	429.79	Ill-defined descriptions and complications of heart disease – Other			✓
		**ICD 10 Codes**			
**Code Type**	**ICD 10 Code**	**ICD 10 Text**	**STEMI**	**NSTEMI**	**MI**
ICD 10 Code	I21	Acute myocardial infarction			✓
ICD 10 Code	I21.0	Acute transmural myocardial infarction of anterior wall	✓		✓
ICD 10 Code	I21.1	Acute transmural myocardial infarction of inferior wall	✓		✓
ICD 10 Code	I21.2	Acute transmural myocardial infarction of other sites	✓		✓
ICD 10 Code	I21.3	Acute transmural myocardial infarction of unspecified site	✓		✓
ICD 10 Code	I21.4	Acute subendocardial myocardial infarction		✓	✓

ICD 10 Code	I21.9	Acute myocardial infarction, unspecified		✓	✓
ICD 10 Code	I22	Subsequent myocardial infarction			✓
ICD 10 Code	I22.0	Subsequent myocardial infarction of anterior wall	✓		✓
ICD 10 Code	I22.1	Subsequent myocardial infarction of inferior wall	✓		✓
ICD 10 Code	I22.8	Subsequent myocardial infarction of other sites	✓		✓
ICD 10 Code	I22.9	Subsequent myocardial infarction of unspecified site		✓	✓
ICD 10 Code	I23	Certain current complications following acute myocardial infarction			✓
ICD 10 Code	I23.0	Hemopericardium as current complication following acute myocardial infarction			✓
ICD 10 Code	I23.1	Atrial septal defect as current complication following acute myocardial infarction			✓
ICD 10 Code	I23.2	Ventricular septal defect as current complication following acute myocardial infarction			✓
ICD 10 Code	I23.3	Rupture of cardiac wall without hemopericardium as current complication following acute myocardial infarction			✓
ICD 10 Code	I23.4	Rupture of chordae tendineae as current complication following acute myocardial infarction			✓
ICD 10 Code	I23.5	Rupture of papillary muscle as current complication following acute myocardial infarction			✓
ICD 10 Code	I23.6	Thrombosis of atrium, auricular appendage, and ventricle as current complications following acute myocardial infarction			✓
ICD 10 Code	I23.8	Other current complications following acute myocardial infarction			✓
ICD 10 Code	I24.1	Dressler syndrome			✓
ICD 10 Code	I25.2	Old myocardial infarction			✓

Table S2. Multivariate COX regression analyses of SHR quintiles and the risk of incident AMI with multiple imputation of five datasets and then pooled.

	Data Set 1	Data Set 2	Data Set 3	Data Set 4	Data Set 5	Pooled results	P- value
AMI							
SHR Q1	1.19 (1.12-1.27)	1.19 (1.12-1.27)	1.20 (1.12-1.27)	1.20 (1.13-1.27)	1.19 (1.12-1.27)	1.20 (1.13-1.27)	< 0.001
SHR Q2	1.16 (1.09-1.23)	1.16 (1.09-1.23)	1.16 (1.09-1.23)	1.16 (1.09-1.23)	1.16 (1.09-1.23)	1.16 (1.09-1.23)	< 0.001
SHR Q3	1.07 (1.0-1.14)	1.07 (1.0-1.14)	1.07 (1.0-1.14)	1.07 (1.0-1.14)	1.07 (1.0-1.14)	1.07 (1.0-1.14)	0.041
SHR Q4	Reference	Reference	Reference	Reference	Reference	Reference	
SHR Q5	1.02 (0.96-1.09)	1.02 (0.96-1.09)	1.02 (0.96-1.09)	1.02 (0.96-1.09)	1.02 (0.96-1.09)	1.02 (0.96-1.09)	0.468
STEMI							
SHR Q1	1.21 (1.08-1.35)	1.21 (1.08-1.35)	1.21 (1.08-1.35)	1.21 (1.08-1.35)	1.21 (1.08-1.35)	1.21 (1.08-1.35)	< 0.001
SHR Q2	1.19 (1.06-1.33)	1.19 (1.06-1.33)	1.19 (1.06-1.33)	1.19 (1.06-1.33)	1.19 (1.06-1.33)	1.19 (1.06-1.33)	0.004
SHR Q3	1.06 (0.94-1.19)	1.06 (0.94-1.19)	1.06 (0.94-1.19)	1.06 (0.94-1.19)	1.06 (0.94-1.19)	1.06 (0.94-1.19)	0.344
SHR Q4	Reference	Reference	Reference	Reference	Reference	Reference	
SHR Q5	0.96 (0.85-1.09)	0.97 (0.85-1.09)	0.97 (0.85-1.09)	0.97 (0.85-1.09)	0.97 (0.85-1.09)	0.97 (0.85-1.09)	0.567
NSTEMI							
SHR Q1	1.24 (1.14-1.35)	1.24 (1.14-1.35)	1.24 (1.14-1.35)	1.24 (1.14-1.35)	1.24 (1.14-1.35)	1.24 (1.14-1.35)	< 0.001
SHR Q2	1.15 (1.06-1.25)	1.15 (1.06-1.25)	1.15 (1.06-1.25)	1.15 (1.06-1.25)	1.15 (1.06-1.25)	1.15 (1.06-1.25)	0.001
SHR Q3	1.10 (1.01-1.20)	1.10 (1.01-1.20)	1.10 (1.01-1.20)	1.10 (1.01-1.20)	1.10 (1.01-1.20)	1.10 (1.01-1.20)	0.037
SHR Q4	Reference	Reference	Reference	Reference	Reference	Reference	
SHR Q5	1.04 (0.95-1.13)	1.04 (0.95-1.13)	1.04 (0.95-1.13)	1.04 (0.95-1.13)	1.04 (0.95-1.13)	1.04 (0.95-1.13)	0.436

The results have been adjusted for age, sex, race, body mass index, Townsend Deprivation Index, physical activity, diet score, insulin use, fasting time, diabetes mellitus, smoking and drinking status.

Table S3. Multivariate COX regression analyses of SHR quintiles and the risk of incident AMI after excluding the participants within two years of follow-up.

**AMI**		
SHR Q1	1.18 (1.10-1.25)	< 0.001
SHR Q2	1.17 (1.10-1.25)	< 0.001
SHR Q3	1.07 (1.0-1.14)	0.05
SHR Q4	Reference	
SHR Q5	1.01 (0.94-1.08)	0.80
**STEMI**		
SHR Q1	1.17 (1.03-1.31)	0.012
SHR Q2	1.21 (1.08-1.37)	0.002
SHR Q3	1.06 (0.95-1.20)	0.371
SHR Q4	Reference	
SHR Q5	0.93 (0.82-1.06)	0.274
**NSTEMI**		
SHR Q1	1.23 (1.13-1.34)	< 0.001
SHR Q2	1.16 (1.06-1.27)	0.001
SHR Q3	1.10 (1.0-1.20)	0.047
SHR Q4	Reference	
SHR Q5	1.03 (0.94-1.13)	0.583

The results have been adjusted for age, sex, race, body mass index, Townsend Deprivation Index, physical activity, diet score, insulin use, fasting time, diabetes mellitus, smoking and drinking status.

Table S4. Multivariate Cox regression analysis of SHR quintiles and AMI risk within the 2.5th–97.5th percentile range of SHR

**AMI**		
SHR Q1	1.17 (1.10-1.25)	< 0.001
SHR Q2	1.14 (1.07-1.21)	< 0.001
SHR Q3	1.07 (1.0-1.14)	0.053
SHR Q4	Reference	
SHR Q5	0.99 (0.93-1.06)	0.832
**STEMI**		
SHR Q1	1.18 (1.05-1.32)	0.005
SHR Q2	1.17 (1.04-1.31)	0.009
SHR Q3	1.05 (0.93-1.19)	0.436
SHR Q4	Reference	
SHR Q5	0.92 (0.81-1.05)	0.215
**NSTEMI**		
SHR Q1	1.22 (1.12-1.33)	< 0.001
SHR Q2	1.13 (1.04-1.24)	0.006
SHR Q3	1.09 (1.0-1.20)	0.05
SHR Q4	Reference	
SHR Q5	1.0 (0.91-1.10)	0.986

The results have been adjusted for age, sex, race, body mass index, Townsend Deprivation Index, physical activity, diet score, insulin use, fasting time, diabetes mellitus, smoking and drinking status.

Table S5. Multivariable Cox regression of SHR quintiles and incident AMI risk with additional adjustment for hypertension, antihypertensive use, and lipid-lowering therapy.

**AMI**		
SHR Q1	1.18 (1.10-1.25)	< 0.001
SHR Q2	1.14 (1.07-1.22)	< 0.001
SHR Q3	1.07 (1.0-1.14)	0.041
SHR Q4	Reference	
SHR Q5	0.99 (0.92-1.05)	0.701
**STEMI**		
SHR Q1	1.19 (1.06-1.34)	0.003
SHR Q2	1.18 (1.04-1.32)	0.007
SHR Q3	1.05 (0.93-1.19)	0.415
SHR Q4	Reference	
SHR Q5	0.92 (0.81-1.04)	0.20
**NSTEMI**		
SHR Q1	1.23 (1.13-1.33)	< 0.001
SHR Q2	1.14 (1.04-1.24)	0.004
SHR Q3	1.10 (1.0-1.20)	0.040
SHR Q4	Reference	
SHR Q5	0.99 (0.91-1.09)	0.896

The results have been adjusted for age, sex, race, body mass index, Townsend Deprivation Index, physical activity, diet score, insulin use, fasting time, diabetes mellitus, hypertension, antihypertensives, lowering lipids therapy, smoking and drinking status.

Figure S1. Directed acyclic graph of the link between SHR and the incidence of AMI.

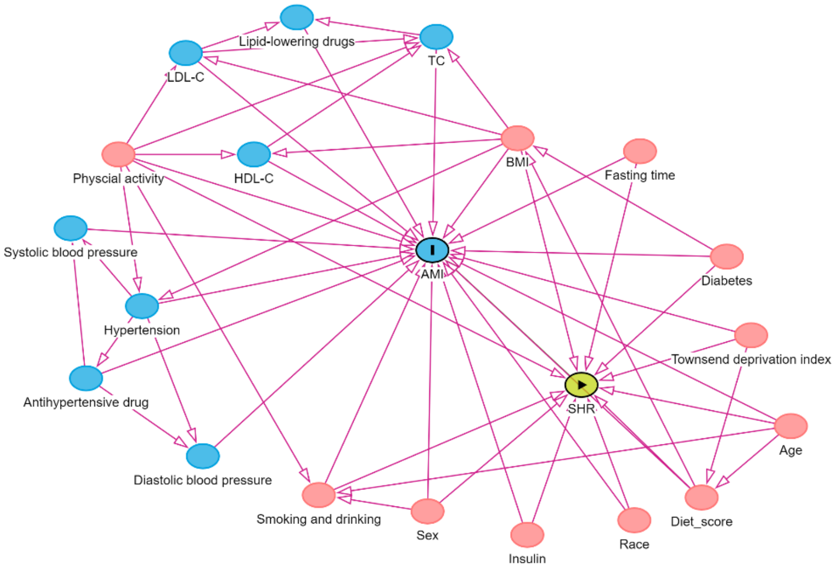

SHR, stress hyperglycemia ratio. AMI, acute myocardial infarction.

Diagram created with the help of DAGitty.net (www.dagitty.net). Minimal adjustment age, sex, race, body mass index, Townsend Deprivation Index, physical activity, diet score, insulin use, fasting time, diabetes mellitus, smoking and drinking status.

Pink represents exposure factors of interest and blue represents outcomes of interest and potential mediators of exposure factor-outcome associations that should not be adjusted for in the main analyses.

Figure S2. Histogram of the distribution of Time to AMI incidence.

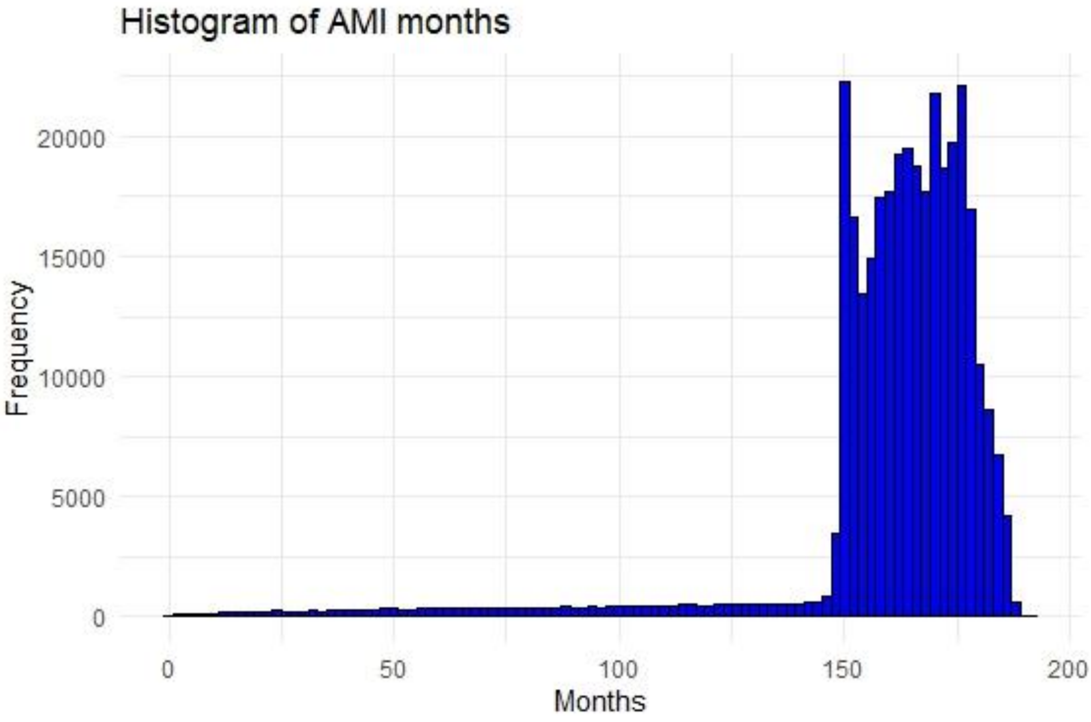

AMI, acute myocardial infarction.

Figure S3. Normal Q-Q Plot of time to AMI incidence.

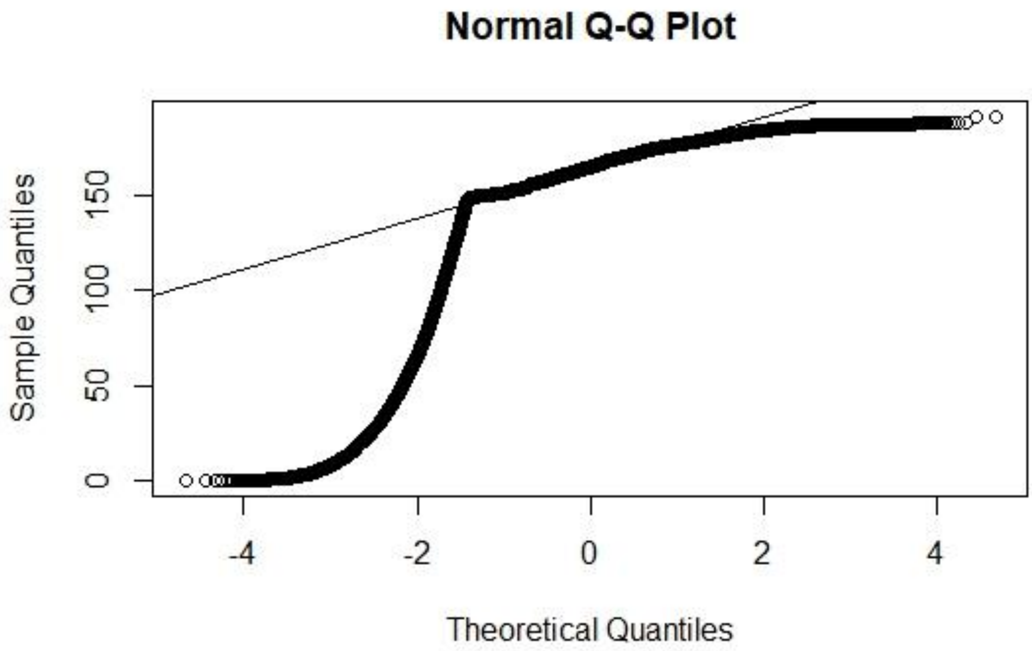

AMI, acute myocardial infarction.

Appendix 1. Calculation of inflection points in threshold effect analysis.

We initially employ restricted cubic splines to assess the nonlinear characteristics of the independent variable and to explore potential partitioning into distinct intervals. Subsequently, segmented regression, also known as piece-wise regression, is applied. This method involves fitting separate line segments to each identified interval. To determine the presence of a threshold effect, a log-likelihood ratio test is conducted, comparing a one-line (non-segmented) model to the segmented regression model. The determination of the inflection point, which connects these segments and is based on maximum likelihood, follows a two-step recursive approach.

The first step involves narrowing down the inflection point to a 10-percentile range of the independent variable. Testing 19 segmented regression models at percentile increments from 5% to 95%, we identify which percentile point yields the highest likelihood. The precise inflection point is then further narrowed down to within +/- 4% percentile of this identified point, termed Kmin and Kmax, respectively.

The second step entails pinpointing the exact inflection point between Kmin and Kmax using the recursive method. This involves initially running three models with inflection points set at Q1 (25th percentile), Q2 (50th percentile), and Q3 (75th percentile) within the Kmin and Kmax range. The model yielding the highest likelihood among these three determines the next range for Kmin and Kmax, which is adjusted to +/- 25% of the corresponding quartile point. This recursive halving process continues until a specific value of the independent variable is identified, which, when used as the inflection point, grants the segmented regression model its highest likelihood.
